# *In vivo* selection of the MDA-MB-231br/eGFP cancer cell line to obtain a clinically relevant rat model for triple negative breast cancer brain metastasis

**DOI:** 10.1371/journal.pone.0243156

**Published:** 2020-12-02

**Authors:** Valerie De Meulenaere, Benedicte Descamps, Olivier De Wever, Christian Vanhove, Karel Deblaere

**Affiliations:** 1 Department of Radiology, Ghent University Hospital, Ghent, Belgium; 2 IBiTech—Medisip—Infinity Lab, Ghent University, Ghent, Belgium; 3 Department of Experimental Cancer Research, Ghent University, Ghent, Belgium; IRCCS Polyclinic San Marino Hospital, ITALY

## Abstract

Young triple negative breast cancer (TNBC) patients are at high risk for developing very aggressive brain metastases associated with a poor prognosis and a high mortality rate. Preclinical models that allow follow-up by magnetic resonance imaging (MRI) can contribute to the development of new therapeutic approaches for brain metastasis. To date, preclinical brain tumor research has almost exclusively relied on xenograft mouse models. Yet, rats are an ideal model for imaging of brain metastasis as their larger brain offers better relative spatial resolution compared to a mouse brain. For the development of a clinically relevant rat model for TNBC brain metastasis, the MDA-MB-231br/eGFP cancer cell line can be used. However, as a result of species-dependent extracranial features, the propensity of the MDA-MB-231br/eGFP cancer cell line to metastasize exclusively to the brain needs to be enhanced by *in vivo* selection. In this study, repeated sequential passages of metastatic cancer cells obtained from brain metastases in nude rats were performed. Brain metastasis formation was evaluated using preclinical MRI, while bone metastasis formation was assessed using high-resolution computed tomography (CT) and 2-deoxy-2-[^18^F] fluoro-D-glucose ([^18^F] FDG) positron emission tomography (PET) imaging. Our results demonstrated that the metastatic tumor burden in the rat brain (number and volume) significantly increased with increasing passage, while the metastatic tumor burden in the skeleton (i.e., number of metastasis-affected bones) significantly decreased with increasing passage. However, bone metastasis development was not reduced to a negligible amount. Consequently, despite *in vivo* selection, our rat model is not recommended for investigating brain metastasis as a single disease. Our findings highlight the importance of well-reasoned selection of both the preclinical model and the cancer cell line in order to obtain reliable and reproducible scientific results.

## Introduction

Brain metastasis poses a severe problem in the treatment of young triple negative breast cancer (TNBC) patients with metastatic disease [1]. TNBC is an aggressive and heterogeneous breast cancer subtype which lacks the common therapeutic targets, making the clinical management of this type of breast cancer particularly challenging. Around 25% of the TNBC patients will develop highly aggressive brain metastasis and the median survival from diagnosis is about 7.3 months [2]. Although significant improvements have been made in treatment and early diagnosis, metastatic brain tumors still remain associated with a poor prognosis and a high mortality rate [3,4]. Brain metastases are routinely diagnosed with contrast enhanced (CE) magnetic resonance imaging (MRI) as it provides excellent soft tissue contrast and spatial resolution resulting in anatomical detail [5].

Preclinical models that allow follow-up with medical imaging techniques provide a non-invasive method to decipher the mechanisms underlying the metastatic process and to develop new therapeutic approaches [6]. Moreover, imaging of small laboratory animals offers the unique opportunity to monitor the entire spectrum of the disease process [7]. For instance, brain metastasis development in the rat brain can be monitored by repeated MRI without the need of sacrificing the animals [8]. Selection of an appropriate preclinical model is crucial and mainly depends on the scientific question being investigated [9]. For brain metastasis research, such a model should reflect the clinical observations and summarize the metastatic process in its dynamic environment [10]. To date, preclinical TNBC brain metastasis research has almost exclusively relied on xenograft mouse models, notwithstanding rats are an ideal model for imaging of brain metastasis. Rats have a larger brain, which offers better relative spatial resolution and clinical deterioration is expected to be less extensive in function of total brain volume.

Our research group previously aimed to establish a clinically relevant rat model for TNBC brain metastasis with the MDA-MB-231br (transduced with eGFP) cancer cell line originally developed by Yoneda and colleagues [11,12]. Unfortunately, early formation of bone metastases was clinically observed and evidenced by 2-deoxy-2-[^18^F]fluoro-D-glucose ([^18^F]FDG) positron emission tomography (PET) and high-resolution computed tomography (CT) [12]. Consequently, this rat model was not suited for the study of brain metastasis as a single disease and associated therapeutic strategies. In order to improve the tropism of the MDA-MB-231br/eGFP cancer cell line to metastasize uniquely to the brain, *in vivo* selection in rats is required [12].

In this study, we attempted to enhance the metastatic propensity of the MDA-MB-231br/eGFP cancer cell line to the brain by repeated sequential *in vivo* selection in nude rats.

## Materials and methods

### Cell culture

The brain metastatic derivative of the TNBC cell line MDA-MB-231 transduced with eGFP (MDA-MB-231br/eGFP) authenticated and free of mycoplasma was maintained as described by Yoneda and colleagues [11].

MDA-MB-231br/eGFP cancer cells were grown in Dulbecco’s modified Eagle’s medium supplemented with 10% fetal calf serum, 1% penicillin-streptomycin antibiotics, 0.0005% fungizone, 1% pyruvate and 1 mg/ml geneticin at 37°C and 10% CO_2_. The MDA-MB-231br/eGFP cancer cell line was regularly tested for Mycoplasma by using the MycoAlert Plus Kit (Lonza).

### Rat model for TNBC brain metastasis

This study protocol was approved by the Ghent University ethics committee for animal experiments (ECD 14/18). All animals were kept and handled according to the European guidelines and housed under environmentally controlled conditions (12 hours normal light/dark cycles, 20°C– 24°C and 40–70% relative humidity) with food and water ad libitum. Animals were fasted overnight before [^18^F]FDG PET scans were performed.

**Fig 1:**
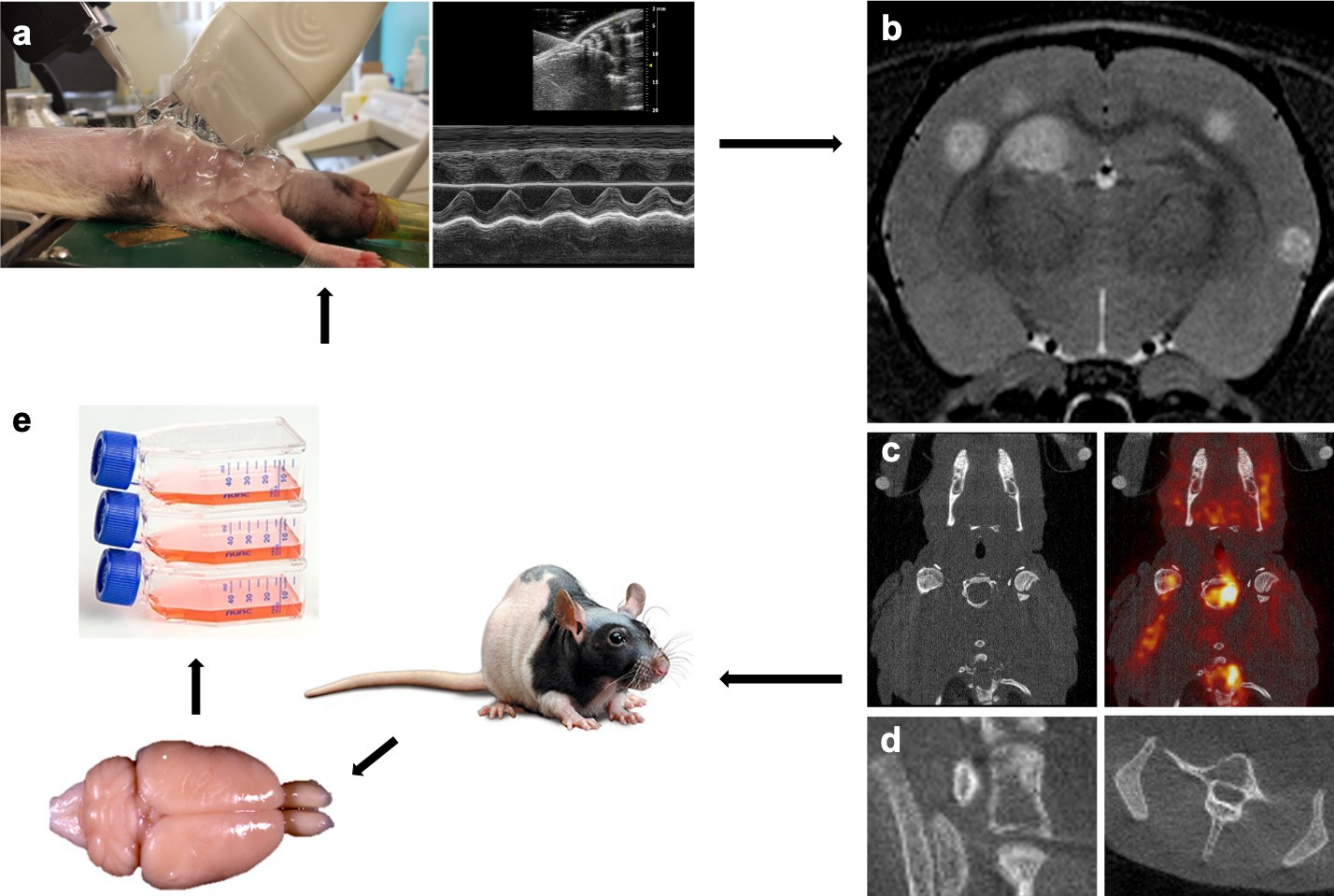
Establishment of the MDA-MB-231br/eGFP subpopulations. a. Intracardiac injection of the MDA-MB-231br/eGFP cancer cells using ultrasound guidance. b-d. Follow-up of metastasis development with multimodal imaging. Preclinical 7 T MRI for assessment of brain metastases (b), [^18^F]FDG PET/CT for visualization of extracranial metastases (c), and high-resolution CT for the detection of bone metastases (d). e. Isolation of the MDA-MB-231br/eGFP cancer cells from the brain metastatic lesions followed by cell culture. This procedure was repeated five times.

Six groups of female nude rats (passage 1 (P1): n = 8, passage 2 (P2): n = 9, passage 3 (P3): n = 8, passage 4 (P4): n = 5, passage 5 (P5): n = 10, passage 6 (P6): n = 8; 5-weeks old, Crl:NIH-Foxn1^rnu^, Charles River) were intracardially injected with 100,000 MDA-MB-231br/eGFP cancer cells using ultrasound guidance to target the left ventricle. For the intracardiac injection, rats were anesthetized using 1.5–2% isoflurane gas mixed with oxygen administered at a flow rate of 0.2 l/min and placed supine with all four limbs fixated on the heated stage. Ultrasonographic gel was applied and an ultrasound probe (FUJIFILM VisualSonics, Vevo 2100, Toronto, Canada) was used to find the left ventricle of the heart. A syringe, secured into a holder, containing a 400 μl suspension of cancer cells was injected slowly after visual confirmation of the needle (3/4-inch-long 27-gauge) in the left ventricle of the heart (Fig 1A). After intracardiac injection, rats were examined by T2 weighted (T2w) MRI to monitor brain metastasis development. Of note, our research group previously compared T2w MRI with CE T1 weighted (T1w) MRI for the detection of brain metastasis. These results indicated that T2w MRI was the better option for detection of brain metastasis in our rat model for TNBC brain metastasis using the MDA-MB-231br/eGFP cancer cell line [12].

### Humane endpoints

Taking the humane endpoints into account, rats were immediately euthanized when clinical or behavioral signs including paralysis, reduced activity, balance problems, absence of grooming, hunched posture or recent weight loss (>20%) was observed. At the end of the experiment or when the humane endpoints were reached, rats were euthanized by an intravenous (IV) injection of pentobarbital (120 mg/kg).

### Multimodal imaging

#### MRI for follow-up of brain metastasis formation

MRI was performed on a 7 T system (PharmaScan 70/16, Bruker, Ettlingen, Germany) to visualize metastasis development in the brain. The rats were anesthetized with 1.5–2% isoflurane and O_2_ and through a nose cone fixed on the Bruker rat restrainer. A heating pad was placed beneath each rat to maintain body temperature at 37°C before it was placed inside the MRI. With the use of T2w MR images (SE RARE, 109 μm in-plane resolution, TR/TE 6346/37 ms) parenchymal metastases were visualized 3, 4, 5 and 6 weeks after intracardiac injection (i.e., post-injection (PI)) of the cancer cells [12] (Fig 1B).

#### [^18^F]FDG PET/CT for extracranial metastasis formation

For our experimental rat model using the MDA-MB-231br/eGFP cancer cell line, the optimal imaging protocol for the detection of extracranial metastases, especially bone metastases, has previously been determined as a [^18^F]FDG PET/CT scan acquired 4–5 weeks PI of the cancer cells [12].

Static whole-body [^18^F]FDG PET/CT (60 minutes acquisition, Triumph-II, Trifoil imaging®, Northridge, USA) was assessed to evaluate potential metastasis development outside the brain. Rats were anesthetized with 1.5–2% isoflurane and O_2_ for the duration of the PET/CT acquisitions. Polyethylene tubing was placed in the lateral tail vein to allow IV injection of 37 MBq [^18^F]FDG. After tracer uptake of 60 minutes, the animals were imaged with their body temperature maintained at 37°C using a heated bed. PET data were reconstructed using a maximum likelihood expectation maximization (MLEM) algorithm with 50 iterations and a reconstructed voxel size of 0.5 × 0.5 × 1.157 mm. A CT acquisition (Triumph-II, Trifoil imaging®, Northridge, USA) was acquired directly after the PET scan, on the same imaging device, for anatomical correlation (Fig 1C).

#### High-resolution CT for visualization of bone metastasis

For the detection of possible bone metastases, full-body spiral high-resolution CT acquisitions (7 minutes acquisition, X-CUBE, MOLECUBES NV, Ghent, Belgium) with 460 μA tube current and 50 kVp tube voltage were performed 4–5 weeks PI. The full body spiral scans were reconstructed using an iterative algorithm (ISRA) with a voxel size of 200 μm (Fig 1D).

### Establishment of the MDA-MB-231br subpopulations

To establish the MDA-MB-231br/eGFP subpopulations, the MDA-MB-231br/eGFP cancer cells from the brain metastatic lesions (preferably from the rat with the lowest number of metastasis-affected bones) were isolated, grown in culture (MDA-MB-231br/eGFP P1), and again inoculated into the left ventricle of the heart of female nude rats. This procedure was repeated five times (Table 1, Fig 1).

**Table 1:** Establishment of the MDA-MB-231br/eGFP subpopulations.

Passage	Inoculated cancer cell line	Isolated cancer cell line	n =
1	MDA-MB-231br/eGFP	MDA-MB-231br/eGFP P1	8
2	MDA-MB-231br/eGFP P1	MDA-MB-231br/eGFP P2	9
3	MDA-MB-231br/eGFP P2	MDA-MB-231br/eGFP P3	8
4	MDA-MB-231br/eGFP P3	MDA-MB-231br/eGFP P4	5
5	MDA-MB-231br/eGFP P4	MDA-MB-231br/eGFP P5	10
6	MDA-MB-231br/eGFP P5	MDA-MB-231br/eGFP P6	8

The brain metastatic lesions were dissociated to a single-cell suspension by enzymatic digestion of the extracellular adhesion proteins and matrix proteins. The tissue was cut into small pieces with a scalpel, then digested enzymatically and further dissociated into a single-cell suspension with the brain tumor dissociation kit with trypsin (Miltenyi Biotec, Leiden, The Netherlands) and the corresponding gentleMACS dissociator programs (Miltenyi Biotec, Leiden, The Netherlands) according to the manufacturer’s instructions.

### Image analysis

Volume and number of brain metastases were assessed on T2w MR images acquired 3 and 4 weeks after intracardiac injection of the cancer cells, when all rats were still alive [12]. Brain metastases’ volumes were measured by manually outlining hyperintense regions on individual slices of T2w MR images using OsiriX software (OsiriX v.5.8.1). The obtained tumor areas were then multiplied by the slice thickness (0.6 mm) to calculate the volume of each brain metastasis.

Bone metastasis development was assessed on high-resolution CT images acquired 4–5 weeks after intracardiac injection through counting the number of metastasis-affected bones.

### Statistical analysis

For the number of brain metastases and the volume (total and average) of brain metastases, linear mixed models were fitted with a random intercept for ‘animal ID’ to account for the repeated measurements within animals. Passage (6 categories, with the sixth passage as reference), timepoint (week 3 and week 4), and their two-way interaction were included as fixed effects. Estimated marginal means were computed for each combination of passage and timepoint together with their corresponding 95% Wald confidence intervals (CIs). These results were visualized in a mean profile plot. Error bars represent the 95% CIs around the estimated arithmetic means. To derive robust 95% CIs of the estimated mean differences (compared to the sixth passage at a certain timepoint), we took 2500 samples using the Wild bootstrap method and requested Bias Corrected and accelerated (BCa) CIs. In addition, linear mixed models were fitted where passage was considered as a continuous covariate and the corresponding estimated regression lines were plotted on top of the mean profile plot.

For the number of metastasis-affected bones two negative binomial models were fitted, once with passage as a categorical fixed effect (with the sixth passage as reference) and once with passage as continuous covariate. The predicted mean number of metastasis-affected bones and corresponding 95% CIs are plotted according to the passage number.

No correction for multiple testing was performed, as Bonferroni correction was considered too strict and conservative. However, when applying Bonferroni correction, p-values should be compared to a significance level of 0.0029 as 17 comparisons were made for each ‘brain’ outcome.

All models were fitted with SPSS version 25. Mean profile plots were made with R (R version 3.6.1).

## Results

### Analysis of brain metastases

**Fig 2:**
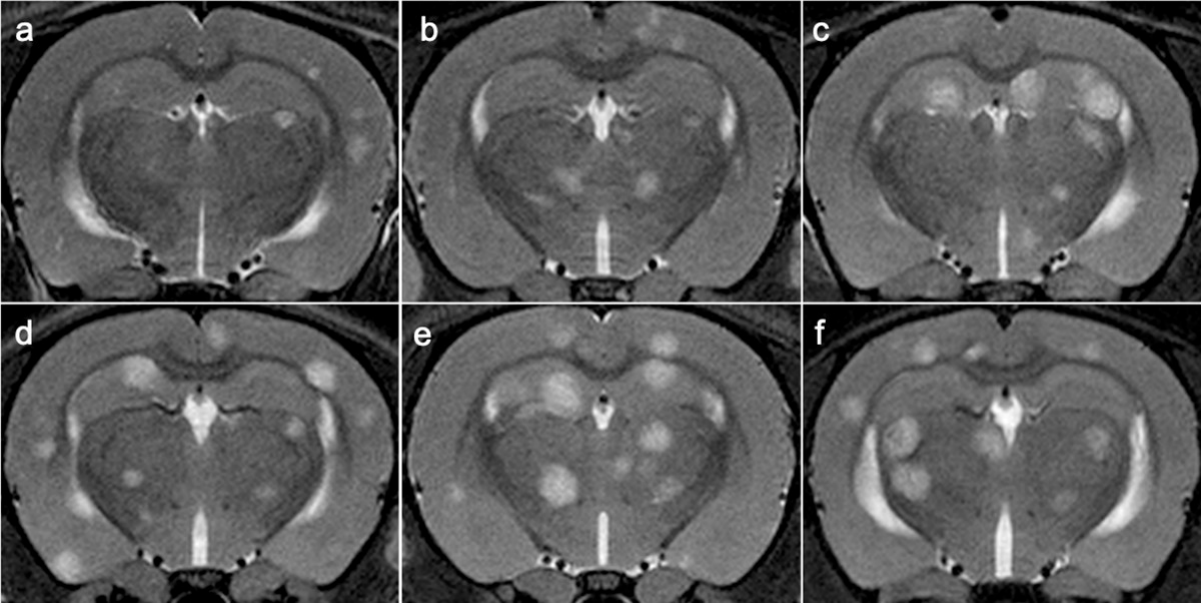
MR imaging of brain metastasis development per passage. a–f. In vivo serial T2w MRI scans showing an increase of brain metastasis development per passage. T2w MR images from a representative case for P1 (a), P2 (b), P3 (c), P4 (d), P5 (e), P6 (f).

**Fig 3:**
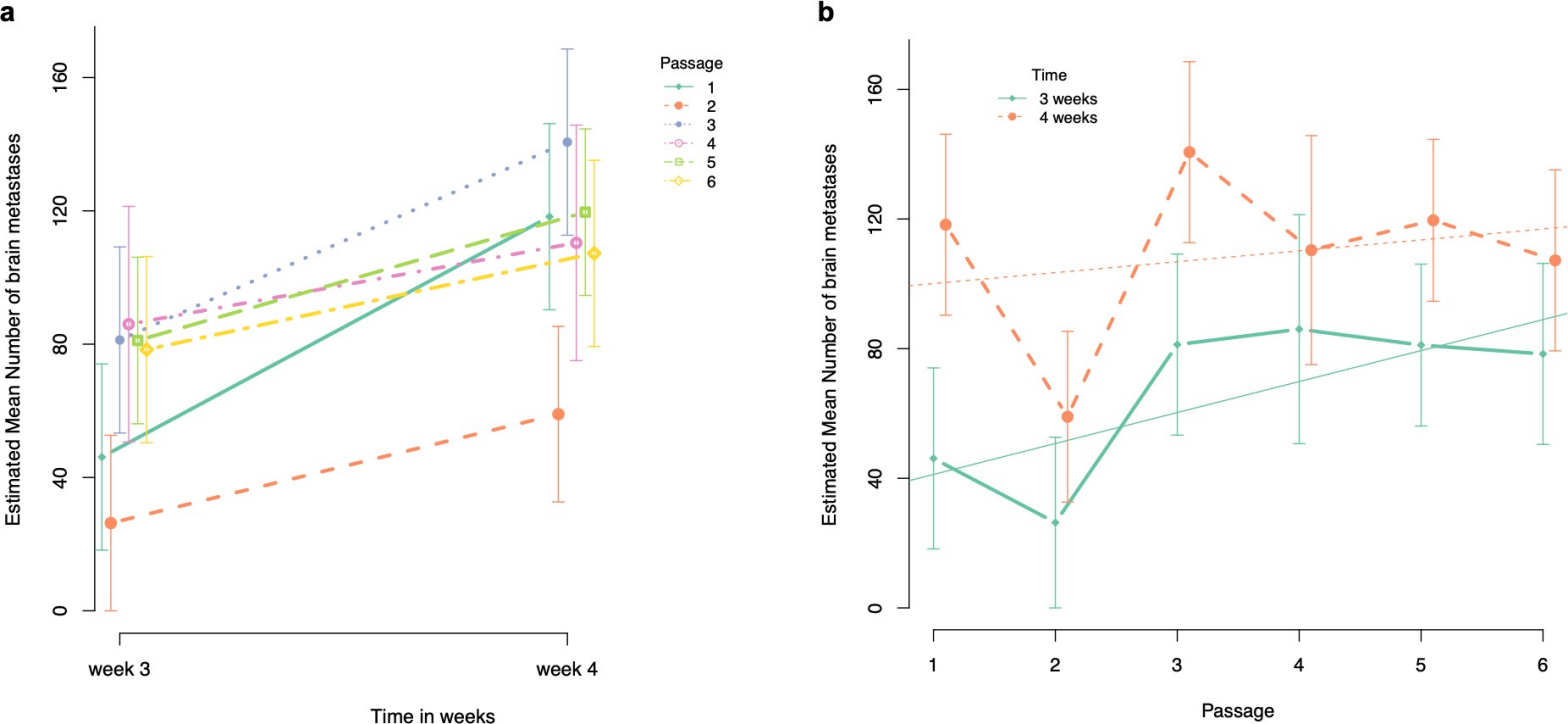
Graphical illustration of the estimated mean number of brain metastases. a. Estimated mean number of brain metastases at week 3 and week 4 after intracardiac injection of the cancer cells for each passage. b. Evolution of the estimated mean number of brain metastases per passage at week 3 and 4 after intracardiac injection of the cancer cells.

**Fig 4:**
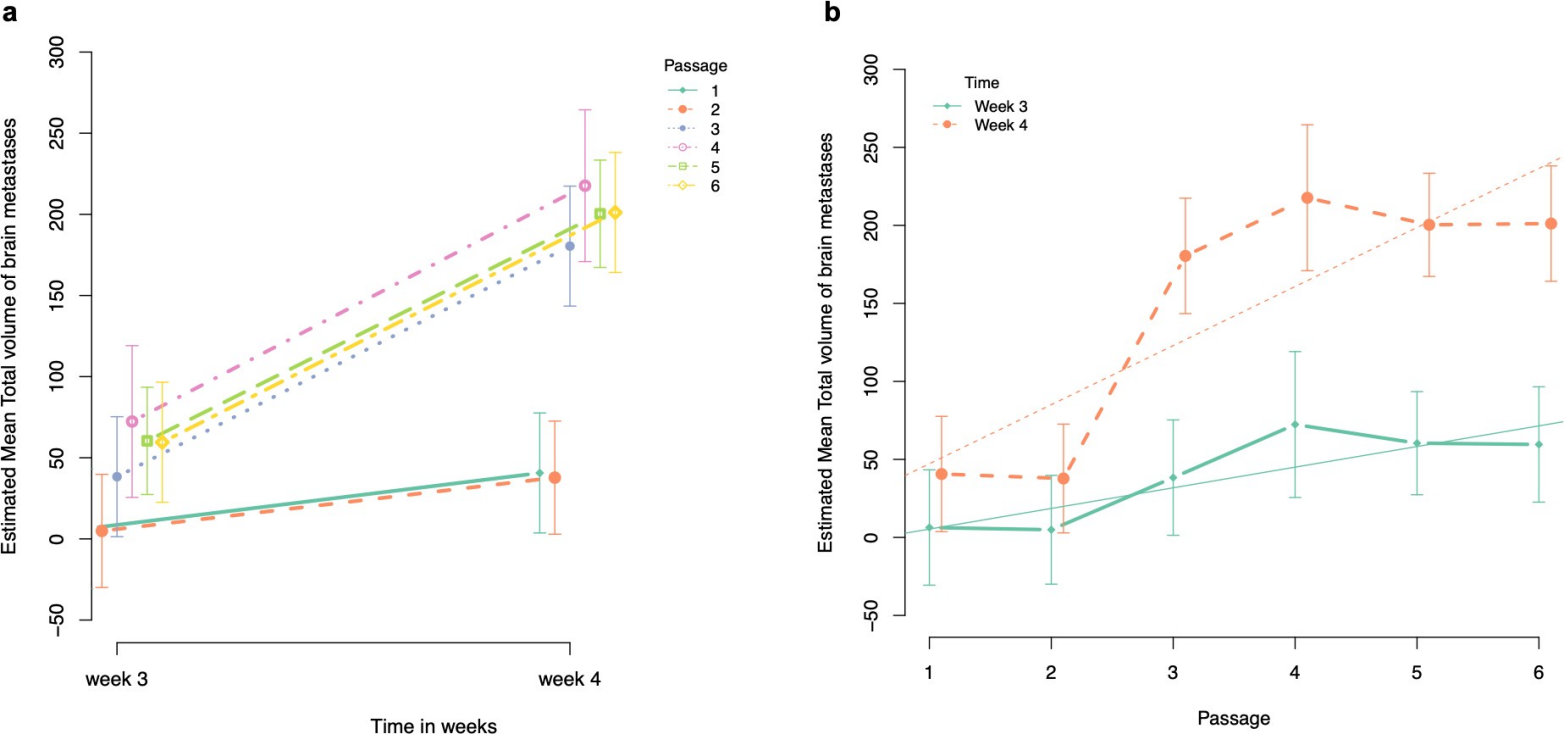
Graphical illustration of the estimated mean total volume of brain metastases. a. Estimated mean total volume of brain metastases at week 3 and week 4 after intracardiac injection of the cancer cells for each passage. b. Evolution of the estimated mean total volume of brain metastases per passage at week 3 and 4 after intracardiac injection of the cancer cells.

**Fig 5:**
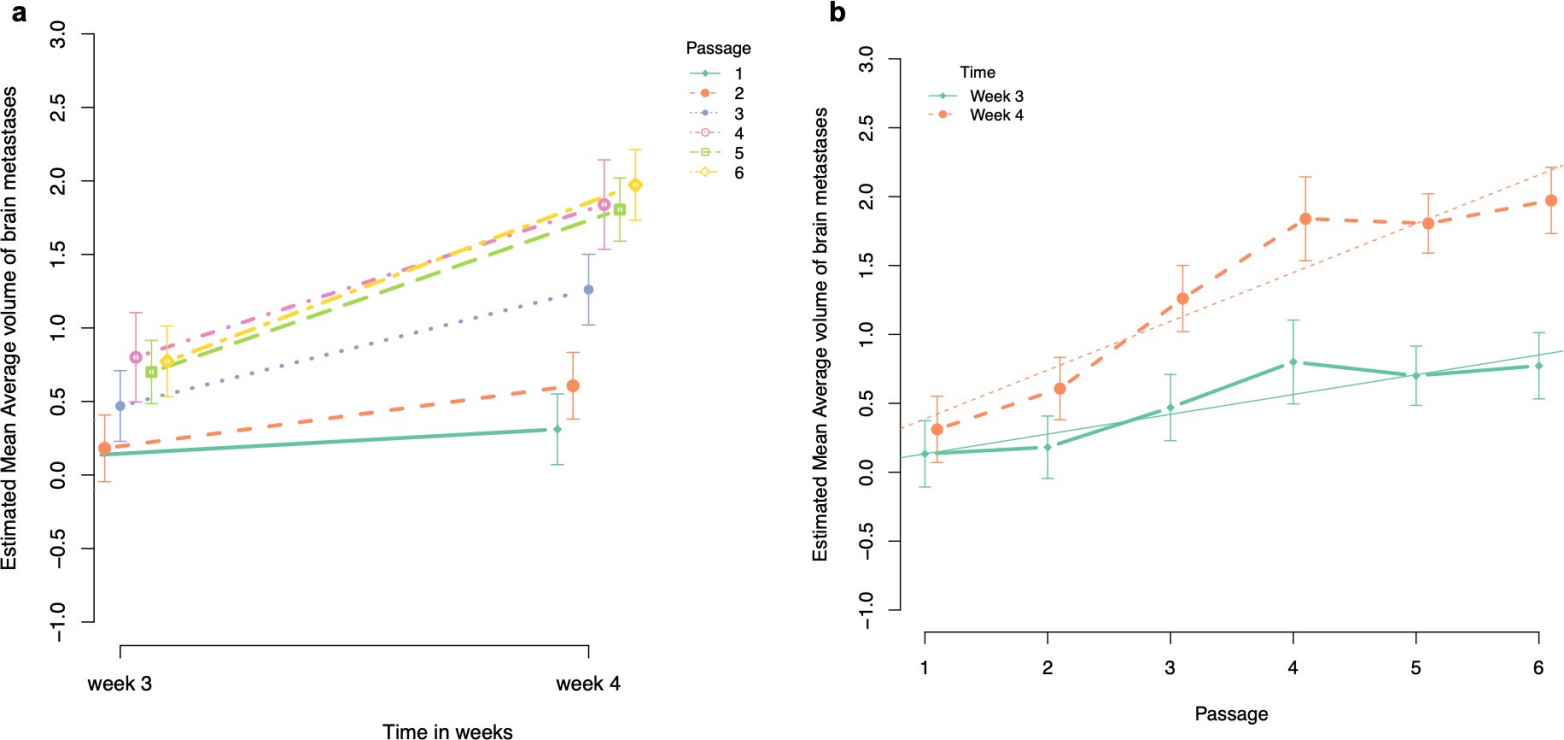
Graphical illustration of the estimated mean average volume of brain metastases. a. Estimated mean average volume of brain metastases at week 3 and week 4 after intracardiac injection of the cancer cells for each passage. b. Evolution of the estimated mean average volume of brain metastases per passage at week 3 and 4 after intracardiac injection of the cancer cells.

T2w images were used to evaluate the tumor burden (i.e., total and average volume, and number of metastatic lesions) in the rat brain (Fig 2). The results of brain metastases analysis are visualized for each passage at week 3 and week 4 after intracardiac injection of the cancer cells (Figs 3–5). Statistical analysis was performed on data acquired at week 3 and week 4 PI, when all animals were still alive.

**Number of brain metastases.** *Pairwise comparison of number of brain metastases at week 3 PI with the sixth passage as reference*. The estimated mean number of brain metastases was significantly lower for passage 1 and 2 compared to passage 6 (estimated mean difference = -32.25, BCa 95% CI goes from -42.43 to -23.65, p < 0.001, and estimated mean difference = -52.04, BCa 95% CI goes from -58.63 to -46.72, p < 0.001, respectively). No other significant differences in estimated mean number of brain metastases were observed compared to passage 6 (Table 2, Fig 3A).

**Table 2:** Pairwise comparison of estimated mean number of brain metastases at week 3 PI with the sixth passage as reference.

Passage	Estimate	Sig. (2-tailed)	Bca 95% CI	
			Lower Bound	Upper Bound
1	-32.250	< 0.001	-42.432	-23.652
2	-52.042	< 0.001	-58.633	-46.718
3	2.875	0.462	-2.507	6.710
4	7.625	0.051	-1.831	12.220
5	2.725	0.813	-8.623	12.725
6	0			

*Pairwise comparison of number of brain metastases at week 4 PI with the sixth passage as reference*. The estimated mean number of brain metastases was significantly lower for passage 2 compared to passage 6 (estimated mean difference = -48.3, BCa 95% CI goes from—62.94 to—32.13, p < 0.001). In contrast, the estimated mean number of brain metastases was significantly higher for passage 3 compared to passage 6 (estimated mean difference = +33.38, BCa 95% CI goes from + 22.33 to + 46.55, p < 0.001). We were unable to observe any other significant differences in estimated mean number of brain metastases at week 4 PI between passage 1, 4 or 5 compared to passage 6 (Table 3, Fig 3).

**Table 3:** Pairwise comparison of the estimated mean number of brain metastases at week 4 PI with the sixth passage as reference.

Passage	Estimate	Sig. (2-tailed)	Bca 95% CI	
			Lower Bound	Upper Bound
1	11.000	0.461	-14.152	37.756
2	-48.250	< 0.001	-62.939	-32.131
3	33.375	< 0.001	-22.334	46.555
4	3.150	0.641	-7.740	16.259
5	12.350	0.299	-3.984	31.265
6	0			

*Comparison of the slopes (i*.*e*., *estimated mean difference of number of brain metastases between week 3 and week 4 PI) with the slope of the sixth passage as reference*. There was a significant difference between the slope of passage 1 compared to the slope of passage 6 with the slope of passage 1 being 43.25 counts larger compared to the slope of passage 6 (i.e., steeper slope for passage 1) (BCa 95% CI for difference in slopes goes from -67.09 to -20.77 p = 0.040). In addition, comparison between the slopes of passage 3 and 6 showed a significant difference, where the slope of passage 3 is 30.5 counts larger compared to the slope of passage 6 (BCa 95% CI for difference in slopes goes from -41.50 to -21.72, p = 0.002). We could not observe any other significant difference of the slopes compared to passage 6 (Fig 3A).

*Evolution per passage*. At week 3 PI, the estimated mean number of brain metastases for passage 1 was 41.18 (95% CI goes from 19.32 to 63.04) and significantly increases with 9.56 per passage (95% CI goes from 2.39 to 16.73, p = 0.01). In contrast, we were unable to find any significant association between estimated mean number of brain metastases and passage number at week 4 PI (Fig 3B).

#### Total volume of brain metastases

*Pairwise comparison of total volume of brain metastases at week 3 PI with the sixth passage as reference*. The estimated mean total volume of brain metastases was significantly lower for passage 1 and 2 compared to passage 6 (estimated mean difference for passage 1 = -53.227, BCa 95% CI goes from -69.434 to -42.738, p < 0.001, and estimated mean difference for passage 2 = -54.663, BCa 95% CI goes from -71.283 to -43.829, p < 0.001, respectively). For passage 3, 4 and 5, we were unable to observe any significant difference in estimated mean total volume of brain metastases compared to passage 6 (Table 4, Fig 4A).

**Table 4:** Pairwise comparison of estimated mean total volume of brain metastases at week 3 PI with the sixth passage as reference.

Passage	Estimate	Sig. (2-tailed)	Bca 95% CI	
			Lower Bound	Upper Bound
1	-53.227	< 0.001	-69.434	-42.738
2	-54.663	< 0.001	-71.282	-43.829
3	-21.251	0.171	-39.588	-9.447
4	12.782	0.359	-4.292	23.783
5	0.829	0.953	-17.651	13.298
6	0			

*Pairwise comparison of total volume of brain metastases at week 4 PI with the sixth passage as reference*. The results of the estimated mean total volume of brain metastases at week 4 PI were similar to the results obtained at week 3 PI. The estimated mean total volume of brain metastases was significantly lower for passage 1 and 2 compared to passage 6 (estimated mean difference for passage 1 = -160.5, BCa 95% CI goes from -206.6 to -107.26, p < 0.001, and estimated mean difference for passage 2 = -163.4, BCa 95% CI goes from -207.5 to -110.8, p < 0.001, respectively). For passage 3, 4 and 5, we could not observe any significant difference in estimated mean total volume of brain metastases compared to passage 6 (Table 5, Fig 4A).

**Table 5:** Pairwise comparison of estimated mean total volume of brain metastases at week 4 PI with the sixth passage as reference.

Passage	Estimate	Sig. (2-tailed)	Bca 95% CI	
			Lower Bound	Upper Bound
1	-160.506	< 0.001	-206.626	-107.264
2	-163.376	< 0.001	-207.521	-110.805
3	-20.712	0.541	-77.043	44.554
4	16.518	0.735	-54.114	97.585
5	-0.798	0.979	-53.327	58.510
6	0			

*Comparison of the slopes (i*.*e*., *estimated mean difference of total volume of brain metastases between week 3 and week 4 PI) with the slope of the sixth passage as reference*. Evaluation of the slopes (as illustrated in Fig 4A) showed a significant difference between passage 1 and passage 6 where the slope of passage 1 is 107.2 counts lower compared to the slope of passage 6 (i.e., steeper slope for passage 6) (BCa 95% CI for difference in slopes goes from 67.9 to 137.1, p < 0.001). In addition, we found a significant difference between the slopes of passage 2 and passage 6 where the slope of passage 2 is 108.7 counts lower compared to the slope of passage 6 (BCa 95% CI for difference in slopes goes from 69.9 to 138.4, p < 0.001). For passage 3, 4 and 5, we were unable to observe any significant difference between slopes compared to passage 6 (Fig 4A).

*Evolution per passage*. At week 3 PI, the estimated mean total volume of brain metastases was 5.33 mm^3^ for passage 1 (95% CI goes from -23.87 to 34.54) and significantly increased with 13.23 mm^3^ per passage (95% CI goes from 3.65 to 22.81, p = 0.007). At week 4 PI, a significant increase of 37.86 mm^3^ per passage was observed (95% CI goes from 28.28 to 47.45, p < 0.001) with 47.20 mm^3^ being the estimated mean total volume of brain metastases for passage 1 (95% CI goes from -17.99 to 76.41) (Fig 4B).

#### Average volume of brain metastases

*Pairwise comparison of average volume of brain metastases at week 3 PI with the sixth passage as reference*. The estimated mean average volume of brain metastases was significantly lower for passage 1 and 2 compared to passage 6 (estimated mean difference = -0.639, BCa 95% CI goes from -0.979 to -0.299, p < 0.001; estimated mean difference = -0.591, BCa 95% CI goes from -0.922 to -0.261, p = 0.001, respectively). For passage 3, 4 and 5, we were unable to observe any significant difference in estimated mean average volume of brain metastases compared to passage 6 (Table 6, Fig 5A).

**Table 6:** Pairwise comparison of estimated mean average volume of brain metastases at week 3 PI with the sixth passage as reference.

Passage	Estimate	Sig. (2-tailed)	Bca 95% CI	
			Lower Bound	Upper Bound
1	-0,639	< 0.001	-0.979	-0.299
2	-0,591	0.001	-0.922	-0.261
3	-0.303	0.079	-0.643	0.037
4	0.028	0.887	-0.360	0.415
5	-0,073	0.656	-0.395	0.250
6	0			

*Pairwise comparison of average volume of brain metastases at week 4 PI with the sixth passage as reference*. The estimated mean average volume of brain metastases was significantly lower for passage 1, 2 and 3 compared to passage 6 (estimated mean difference between passage 1 and 6 = -1.7, BCa 95% CI goes from -2.0 to -1.3, p < 0.001; estimated mean difference between passage 2 and 6 = -1.4, BCa 95% CI goes from -1.7 to -1.0, p < 0.001; and estimated mean difference between passage 3 and 6 = -0.7, BCa 95% CI goes from -1.1 to -0.3, p = 0.003). For passage 4 and 5, we were unable to observe any significant difference in estimated mean average volume of brain metastases compared to passage 6 (Table 7, Fig 5A).

**Table 7:** Pairwise comparison of estimated mean average volume of brain metastases at week 4 PI with the sixth passage as reference.

Passage	Estimate	Sig. (2-tailed)	Bca 95% CI	
			Lower Bound	Upper Bound
1	-1.662	< 0.001	-1.962	-1.318
2	-1.366	< 0.001	-1.679	-1.011
3	-0.712	0.003	-1.084	-0.326
4	-0.134	0.682	-0.628	0.404
5	-0.167	0.562	-0.582	0.251
6	0			

*Comparison of the slopes (i*.*e*., *estimated mean difference of average volume of brain metastases between week 3 and week 4 PI) with the slope of the sixth passage as reference*. Comparison of the slopes among passages revealed a significant difference between passage 1 and 6, with the mean difference being 1.02 counts lower for passage 1 compared to passage 6 (BCa 95% CI for difference in slopes goes from 0.70 to 1.30, p < 0.001). The slope of passage 2 was also significantly different compared to the slope of passage 6, with the slope of passage 2 being 0.77 counts lower compared to passage 6 (BCa 95% CI for difference in slopes goes from 0.45 to 1.06, p = 0.001). For passage 3, 4 and 5, we were unable to observe any significant difference between the slopes compared to passage 6.

*Evolution per passage*. At week 3 PI, the estimated mean average volume of brain metastases for passage 1 was 0.13 mm^3^ (95% CI goes from -0.05 to 0.31), and significantly increased with 0.14 mm^3^ per passage (95% CI goes from 0.01218 to 0.20, p < 0.001). At week 4 PI, the estimated mean average volume of brain metastases for passage 1 was 0.39 mm^3^ (95% CI goes from 0.30 to 0.41, p < 0.001), and significantly increased with 0.35 mm^3^ per passage (95% CI goes from 0.30 to 0.41, p < 0.001) (Fig 5B).

### Number of metastasis-affected bones

**Fig 6:**
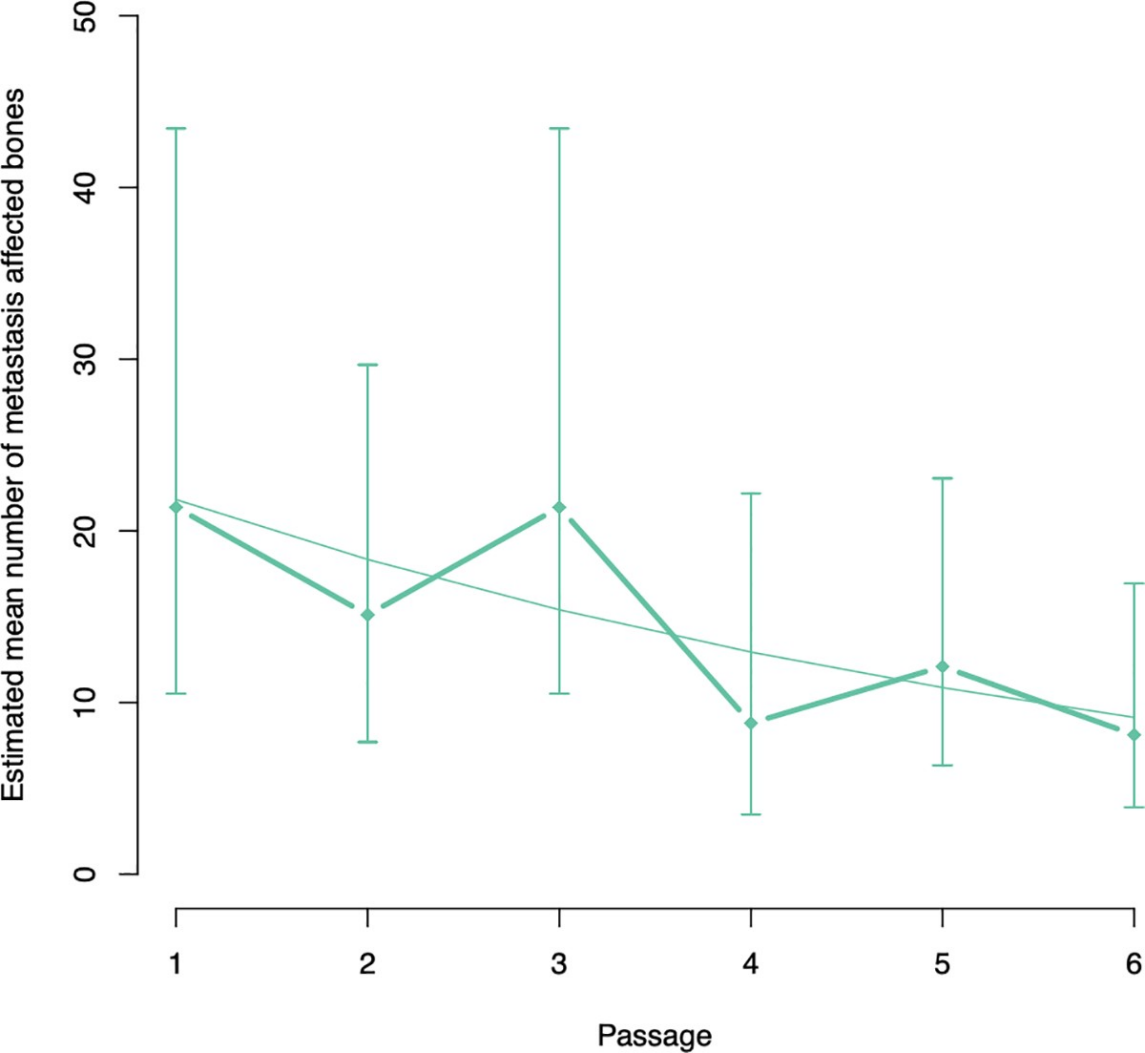
Graphical illustration of the evolution of the estimated mean number of metastasis-affected bones per passage.

We were unable to find any significant difference in estimated mean number of metastasis-affected bones between passage 1, 2, 3, 4 or 5 compared to passage 6 (Table 8). However, the estimated mean number of metastasis-affected bones decreased significantly with 16% per passage (95% CI goes from -0.2% to—29.3%, p = 0.048) (Fig 6).

**Table 8:** Pairwise comparison of estimated mean number of metastasis-affected bones with the sixth passage as reference.

Passage	Exp(B)	Sig. (2-tailed)	Bca 95% CI for Exp(B)	
			Lower Bound	Upper Bound
1	2.631	0.063	0.948	7.301
2	1.860	0.223	0.686	5.041
3	2.631	0.063	0.948	7.301
4	1.083	0.895	0.332	3.528
5	1.489	0.424	0.560	3.957
6	1			

## Discussion

To date, the pathophysiology of brain metastasis and preclinical validation of therapeutic approaches has almost exclusively relied on xenograft mouse models. Until recently, intracardiac injection of mice with brain-seeking cancer cells derived from MDA-MB-231 (TNBC) and BT474 (HER2+) breast cancer cell lines are the most frequently used models for breast cancer brain metastasis as they produce brain metastases at high frequencies [13,14]. Nevertheless, rats are an excellent model for brain metastasis imaging as their larger brain offers better relative spatial resolution. Therefore, our research group previously aimed at developing a clinically relevant rat model for TNBC brain metastasis with the MDA-MB-231br/eGFP cancer cell line [11,12]. Unfortunately, early bone metastasis was clinically observed and evidenced by PET/CT. The bones lesions caused severe clinical symptoms leading to early euthanasia of several rats. Consequently, this rat model was not suited for investigating brain metastasis as a single disease and testing associated therapeutic strategies [12]. In order to reduce the amount of metastatic bone lesions to a negligible amount, the tropism of the MDA-MB-231br/eGFP cancer cell line to metastasize uniquely to the brain needs to be enhanced.

For this purpose, *in vivo* selection in rats has been performed in the present study. Brain metastasis development was assessed with preclinical MRI because of its noninvasive nature, high spatial resolution (up to 50 μm at 7 T) and high signal-to-noise ratio [15]. MRI allows longitudinal follow-up and is an excellent tool for the study of tumor growth. In this study, brain metastasis development (i.e., total and average volume, and number of metastatic lesions) was evaluated on T2w images acquired 3 and 4 weeks PI based on the imaging protocol previously described by our research group (Table 9 gives an overview of the p-values) [12]. At week 3 PI, the estimated mean number of brain metastases was significantly lower for passage 1 and 2 compared to passage 6. Moreover, we observed that the estimated mean number of brain metastases significantly increased with 9.56 per passage. At week 4 PI, the estimated mean number of brain metastases was significantly lower for passage 2 compared to passage 6, but the estimated mean number of brain metastases was significantly higher for passage 3 compared to passage 6. In addition, we were unable to find a significant association between estimated mean number of brain metastases and passage number. This inconsistent observation for estimated mean number of brain metastases at week 4 PI might be the result of the complex relation between number and volume of brain metastases. Percy et al. reported that animals with fewer metastases more often developed larger metastases after inoculation with the MDA-MB-231br cancer cell line [16]. Hence, estimated mean number of brain metastases alone is not an ideal representation for tumor load in the brain. In addition, we evaluated the total and average volume of the brain metastases. The estimated mean total volume of brain metastases was found to be significantly lower for passage 1 and 2 compared to passage 6 at both week 3 and week 4 PI. Moreover, we observed a significant association between estimated mean total volume of brain metastases and number of passage where the estimated mean total volume of brain metastases increased with 13.23 mm^3^ per passage at week 3 PI and with 37.86 mm^3^ per passage at week 4 PI. A similar result was observed for the estimated mean average volume of brain metastases. At week 3 PI, the estimated mean average volume of brain metastases was significantly lower for passage 1 and 2 compared to passage 6. Along with passage 1 and 2, the estimated mean average volume of brain metastases for passage 3 was also significantly lower compared to passage 6 at week 4 PI. Moreover, we observed a significant indication for an increasing estimated mean average volume of brain metastases with 0.14 mm^3^ per passage at week 3 PI and 0.35 mm^3^ per passage at week 4 PI. These observations indicate that the metastatic tumor load in the brain significantly increased with increasing passage. For the evaluation of tumor burden in the skeleton of the rat, the number of metastasis-affected bones was assessed on high-resolution CT images acquired 4–5 weeks PI for each passage. Statistical analysis revealed that the estimated mean number of metastasis-affected bones decreased significantly with 16% per passage (Table 8), as we had expected. Although the estimated mean number of metastasis-affected bones continued to decrease per passage, it was never reduced to an amount that was clinically negligible as previously reported for mice by our research group. We observed and evidenced one metastasis-affected bone (the skull) in two out of five mice [12].

**Table 9:** Overview of p-values with the sixth passage as reference.

Passage	Number of brain metastases		Total volume of brain metastases		Average volume of brain metastases		Number of affected bones
	Week 3	Week 4	Week 3	Week 4	Week 3	Week 4	Week 4
1	<0.001	0.461	<0.001	<0.001	<0.001	<0.001	
2	<0.001	<0.001	<0.001	<0.001	0.001	<0.001	
3	0.461	<0.001	0.171	0.541	0.079	0.003	
4	0.051	0.641	0.359	0.735	0.887	0.682	
5	0.813	0.299	0.953	0.979	0.656	0.562	
6							
Evolution	Sign. increase (0.01)		Sign. increase (0.007)	Sign. increase (<0.001)	Sign. increase (<0.001)	Sign. increase (<0.001)	Sign. decrease (0.048)

Our findings are different to those obtained by Yoneda and colleagues [11]. They developed a brain-seeking clone of the MDA-MB-231 cell line (MDA-MB-231br) by performing repeated sequential passages of metastatic cancer cells obtained from brain metastases in nude mice. The resulting MDA-MB-231br cancer cell line has been described to exclusively disseminate to the mouse brain as no bone metastatic lesions were confirmed with radiographic analysis [11]. Radiography is generally not used as a screening method for bone metastatic lesions because of its poor sensitivity. Several studies have revealed that bone destruction of 50% or more is required for appropriate radiographic detection of bone lesions [17–21]. We assessed high-resolution CT for the detection of the metastatic bone lesions, which is more sensitive compared to radiography [12,20]. CT exhibits superior anatomical detail and distinguishes between different densities, even allowing detection of metastases within the bone marrow before bone destruction has appeared [20].

Of note, these xenograft animal models established by intracardiac injection of brain-seeking cancer cells do not completely represent the heterogeneity of breast cancer and their metastasis [13,14]. In order to provide a better representation of the original tumors of the patients, patient-derived xenograft (PDX) models can be used. However, PDX models for brain metastasis may also develop ‘undesired’ metastasis to other organs resulting in clinical symptoms and hampering long-term follow-up [13,14,22].

In conclusion, we report that the metastatic tumor burden in the rat brain significantly increased with increasing passage, while the metastatic tumor burden in the skeleton significantly decreased with increasing passage. Unfortunately, we were unable to reduce bone metastasis formation to a negligible amount after *in vivo* selection. Therefore, this rat model using the MDA-MB-231br/eGFP is not ideal for investigating brain metastasis as a single disease and testing associated therapeutic strategies. Our observations highlight the importance of well-reasoned selection of the preclinical model and the cancer cell line in order to obtain reliable and reproducible scientific results.

## Supporting information

S1 File The ARRIVE Guidelines Checklist. Completed ARRIVE Guidelines Checklist for reporting animal data in this manuscript.
(PDF)
